# Lost voices

**DOI:** 10.7554/eLife.06536

**Published:** 2015-04-13

**Authors:** Eve Marder

**Affiliations:** Department of Biology and the Volen National Center for Complex Systems, Brandeis University, Waltham, United Statesmarder@brandeis.edu

**Keywords:** living science, careers in science, history of science

## Abstract

When a scientist dies too early in their career we miss them as a colleague and as a person and, as **Eve Marder** explains, we also lose the science they would have done.

When Janis Joplin, John Lennon and Amy Winehouse died, we experienced the pain of losing a special talent too young. Joplin, Lennon and Winehouse, whether you like their music or not, were special. Each had a voice, both as a sound and as a songwriter, that was unique. When they died, we lost their presence as artists of their time and place, and we also lost all of their future music. We can only wonder what they would have sounded like, and what they would have made of today's world. As I hear an old Joplin song or a newer Winehouse song, I never fail to reflect on the songs they never had the time to write.

In contrast, Bob Dylan, Paul McCartney and Bruce Springsteen have aged with us, and we can hear the effects of time on their vocal cords. Just recently I happened to listen to an amazing version of ‘Girl From the North Country’ sung by Dylan and Johnny Cash: the melodic clarity of the young Dylan's voice in that recording exists in stark contrast with how he sounds today. We also see the impact of decades of living on both their music and lyrics. In so doing, we can measure the loss of innocence, the changes in political realities, and the toll that living as a larger than life voice can take on an individual.

**Figure 1:**
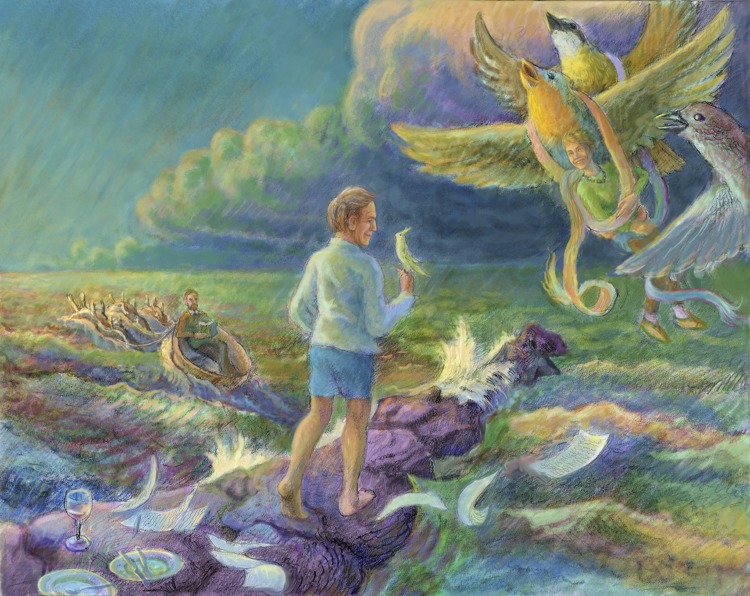
As scientists we have the privilege of knowing our colleagues as people who are often as fascinating and compelling as their work.

ILLUSTRATION: BEN MARDER

We also lose the voices of our scientific colleagues. Sometimes it is in the fullness of time, and we can see how their entire scientific opus developed, how their perspectives, visions and contributions evolved with those of their fields around them. We watch some of them switch fields, and some of them doggedly making stunning contributions over 50 or more years. Just as one can regret and deeply mourn the missing voice of a parent or grandparent, one can regret and mourn the loss of a major figure in the field when they are no longer with us. My undergraduate thesis advisor, Andrew Szent-Györgyi, recently died at the age of 90, and I find myself occasionally echoing his voice and passing on to young students comments he made to me 30 and 40 years ago.

It is a different kind of loss when colleagues die too early in their scientific careers. We miss their vitality and their presence, but we also lose knowing the wonderful science they would have done. Each of us mourns people who had special voices and creative visions of and for science. I miss Peter Getting, who was the acknowledged leader in my field, that of understanding small neuronal circuits. Before he was incapacitated by a major stroke in the late 1980s, while still only in his 40s, he was using the nervous system of the marine mollusk, *Tritonia*, to explore how neuronal dynamics can be understood in terms of their component processes. Not infrequently I wonder what he would be thinking, saying and doing now. I feel sorry that today's students never had the chance to know his quick wit and his insightful mind.

When a plane crash killed Walter Heiligenberg at the peak of his career in 1994, we lost a gentle but fierce advocate for neuroethology. Walter turned the study of the jamming avoidance response in weakly electric fish into a celebratory song of the wonders of how animals behave in the world. Walter's science was rigorous but his support of his colleagues was unfailingly generous, his voice and smile teaching that an uncompromising search for the truth in the natural world should bring us joy.

In October 2014 we lost Allison Doupe. Allison was a wonderful scientist who spent most of her career working on the neurobiological mechanisms of bird song production and learning. While we celebrate and remember her extraordinary accomplishments, we regret the science she never had the chance to do and the discoveries she never made. But also, those of us who mourn Allison grieve for the loss of a woman who took her scientific voice and spoke her mind while retaining true personal grace, dignity and perspective. When I told someone who hadn't heard that she had died, I watched his face crumble with the sadness that one sees only when one regrets the loss of a special person.

Peter, Walter and Allison had unique voices that we lost too early, and all three made singular contributions to understanding the biology of animals in their worlds. As did so many others, too numerous to mention here, people deeply treasured and mourned by some of you and unknown to others of us. Of course there are objective realities about how biological systems work that we all hope to uncover. But the paths of discovery through the unknown are infinitely variable, and the act of remembering the accomplishments of individual scientists is a reminder that creative scholars often find unusual routes to a deeper understanding of the world around us. Their voices, written and spoken, let us all share in the outcome of their unique acts of scientific artistry. As technology moves us quickly forward, it is all the more important to remember that some problems require creative insight from thoughtful individuals.

My students often ask me how, when and where I first met many of the scientists whom I knew or know. Sometimes I can't recall where or when I first met one of my colleagues (although with some I have vivid recollections of our first encounters). A few I have known since graduate school. Others, I have known for 20, 30 or 40 years, although we never worked in the same institution. Dylan and Springsteen are known to me only through their public personas and musical voices. In contrast, as scientists we have the privilege to know our colleagues through their scientific voices as they compose their songs of science, as well as knowing them as people who are often as fascinating and compelling as their work.

